# Real-world reduction in nightmare frequency and distress among San Francisco first responders treated with a prescription wearable device: a 60-day open-label pilot

**DOI:** 10.3389/frsle.2026.1941077

**Published:** 2026-09-09

**Authors:** Brian D. Robertson, Emily F. Wargo, Joel Fay

**Affiliations:** 1 NightWare, Inc., Minneapolis, MN, United States; 2 First Responder Support Network, San Rafael, CA, United States

**Keywords:** first responders, nightmare disorder, nightmares, post-traumatic stress disorder, sleep, wearable device

## Abstract

**Objective:**

Nightmares are a common, distressing condition among individuals with post-traumatic stress disorder (PTSD) and are associated with impaired sleep quality and elevated suicide risk. First responders carry a substantial burden of occupational trauma and PTSD yet are under-represented in nightmare-treatment research. NightWare is an FDA-authorized prescription wearable that monitors heart rate and movement during sleep and delivers vibrotactile feedback intended to interrupt nightmares and improve sleep quality. We evaluated the real-world response to NightWare among San Francisco first responders.

**Methods:**

First responders (law enforcement and fire service) prescribed NightWare in routine clinical care completed structured self-report assessments at Day 0, 30, and 60. First responders were aged 35–65, half were male and half were female. They had a PTSD diagnosis and at least 3 nightmares per week. The co-primary endpoints were nightmare frequency (self-reported nights with nightmares per week, 0–7) and nightmare-related distress (5-point ordinal scale, 0–4). Change was assessed with paired *t*-tests (with Cohen's d) and one-way repeated-measures analysis of variance (RM-ANOVA).

**Results:**

Of 11 first responders who initiated treatment, 10 (91%) completed all assessments. Mean nightmare frequency decreased from 5.2 to 3.6 nights/week [mean change −1.6; 95% CI −2.7 to −0.5; *t*_(9)_ = −3.21, *p* = 0.011; *d* = 1.0] and mean distress decreased from 2.9 to 1.3 [mean change −1.6; 95% CI −2.2 to −1.0; *t*_(9)_ = −6.00, *p* = 0.0002; *d* = 1.9]. RM-ANOVA confirmed a significant decline across the three time points for both endpoints (frequency *p* = 0.009; distress *p* = 0.003). Seven of ten reduced nightmare frequency and nine of ten improved on distress; no participant worsened on either measure. Self-reported sleep quality and treatment satisfaction improved. The participants in this survey used the NightWare device a mean of 76% of nights (45.6 days of the 60-day survey time period). The range of usage was 55–100% (33–60 days) of the study nights.

**Conclusions:**

In this small, open-label, real-world pilot, treatment with NightWare was associated with clinically meaningful reductions in nightmare frequency and distress and high patient acceptability among first responders. These findings are hypothesis-generating; controlled studies with validated instruments are warranted.

## Introduction

Nightmare disorder is a prevalent parasomnia defined by repeated, well-remembered dysphoric dreams that typically involve threats to survival, security, or physical integrity (American Psychiatric Association, 2013). It is frequently comorbid with mental health conditions, particularly post-traumatic stress disorder (PTSD), anxiety, and depression. Clinically significant nightmares—generally defined as nightmares occurring one or more times per week—are common in the general adult population, and any recent nightmares are reported by roughly one in five adults (American Psychiatric Association, 2013; Bjorvatn et al., 2010). The burden is far greater in trauma-exposed populations: frequent, trauma-related nightmares occur in an estimated 50 to 70% of individuals with PTSD, and frequent nightmares are present in approximately half of combat veterans with PTSD compared with only a small minority of those without PTSD (Creamer et al., 2018; Neylan et al., 1998; van Liempt et al., 2013).

First responders—law enforcement officers, firefighters, paramedics, and emergency medical technicians—are repeatedly exposed to potentially traumatic events in the course of their work, and a systematic review and meta-regression estimated the worldwide current prevalence of PTSD among rescue workers at approximately 10%, several-fold higher than the general population (Berger et al., 2012). This population also experiences a disproportionate burden of suicidal thoughts and behaviors, with occupational stress, PTSD, and sleep disturbance among the recurrent contributors (Stanley et al., 2016). Nightmares themselves are an independent predictor of suicide: in a large prospective general-population cohort, frequent nightmares were associated with roughly twice the risk of death by suicide, an association that persisted after adjustment for other factors (Tanskanen et al., 2001). These observations establish nightmare reduction as a clinically meaningful objective in first responders. We emphasize that the present study did not assess suicidality and makes no claim that the intervention reduces suicide risk; this literature is cited only to frame why treating nightmares matters in this population.

The scale of this burden is increasingly well characterized. In a cross-sectional study of 242 active-duty first responders across the United States, over half (53.4%) reported trauma-related nightmare distress within the past week, with 23.2% reporting clinically significant distress; more than one-third (37.8%) screened positive for provisional insomnia and 23.1% for provisional PTSD (Reffi et al., 2023). Participants reported extensive cumulative trauma exposure (a mean of approximately 12 lifetime traumatic event types), dominated by death-threat traumas such as sudden violent death, reflecting the occupational exposure profile of public safety work (Reffi et al., 2023). Notably, trauma-related nightmares and hyperarousal symptoms were independently associated with fear of sleep—a fear of being vulnerable while sleeping—even after adjusting for remaining PTSD symptoms, insomnia, sex, and years of service, suggesting that nightmares are not merely a marker of distress but may actively drive maladaptive sleep-avoidance behaviors that perpetuate insomnia and compound occupational impairment (Reffi et al., 2023). Screening studies suggest the burden of posttraumatic stress symptomatology in the fire service may be even higher than diagnosed prevalence, with 17–22% of urban firefighters meeting screening criteria for clinically significant posttraumatic stress symptoms (Corneil et al., 1999). Among 1,027 current and retired firefighters, career prevalence of suicidal ideation (46.8%) and suicide attempts (15.5%) far exceeded general-population lifetime rates (Stanley et al., 2015). The fire service's own leadership has identified cumulative stress, disrupted sleep, and nightmares as core behavioral health concerns, while acknowledging a culture of stigma that delays help-seeking (International Association of Fire Chiefs, Volunteer and Combination Officers Section, 2017). Untreated nightmares in this population therefore represent both a prevalent clinical problem and a modifiable contributor to a cascade ending in insomnia, functional impairment, and elevated suicide risk.

Effective treatments for nightmare disorder exist but are constrained by access and tolerability. Imagery rehearsal therapy is recommended by the American Academy of Sleep Medicine as a first-line behavioral treatment (Morgenthaler et al., 2018), but qualified providers are scarce and a substantial proportion of patients cannot or will not engage in the therapy (Cook et al., 2010). Prazosin, an alpha-adrenergic antagonist long used for PTSD-related nightmares, did not separate from placebo on sleep quality or distressing dreams in the largest and most rigorous multicenter trial conducted to date, tempering enthusiasm for its routine use (Stanley et al., 2015; Raskind et al., 2018). For first responders specifically, occupational and fitness-for-duty considerations can further limit the use of centrally acting medications. There is therefore a need for safe, accessible, non-pharmacologic options.

NightWare (NightWare, Inc., Minneapolis, MN, https://www.nightware.com) is a prescription digital therapeutic authorized by the U.S. Food and Drug Administration through the De Novo pathway in 2020 for the reduction of sleep disturbance related to nightmares in adults with nightmare disorder or PTSD-associated nightmares (Food U. S. and Drug Administration, 2020). The system consists of a pre-provisioned Apple Watch and paired iPhone prescribed as a dedicated therapeutic kit. During sleep, the watch's heart rate sensor, accelerometer, and gyroscope feed a proprietary algorithm that constructs a personalized “stress index,” with the intervention threshold adaptively updated from the prior 1,000 min of recorded sleep data. When the stress index exceeds threshold—indicating autonomic arousal consistent with a nightmare—the watch delivers vibrotactile stimulation intended to interrupt the arousal without fully waking the patient. Haptic feedback is delivered by the Apple Watch itself, following an escalating three-level protocol: at the lowest intensity, three sets of two quarter-second taps separated by half-second pauses; at medium, three sets of three quarter-second taps; at the highest, five sets of three, with each intervention lasting approximately 3–5 s. If the stress index remains elevated, intensity escalates stepwise; once it falls below threshold, intensity resets. Because the watch and phone are provisioned as a dedicated therapeutic system rather than the patient's personal devices, the watch receives no text, email, or other notifications, so haptic stimulation during sleep is attributable solely to the NightWare intervention (Food U. S. and Drug Administration, 2020). In a randomized, sham-controlled trial of 65 military veterans, both active and sham groups improved, the prespecified between-group comparison did not reach significance, and a *post-hoc* analysis restricted to participants who used the device at least half of nights showed significantly greater improvement with active treatment on sleep quality and nightmare measures (Davenport and Werner, 2023). A subsequent large real-world retrospective assessment of active-duty service members reported that 92.9% experienced at least some improvement in sleep and nightmares with good tolerability (Robertson et al., 2023). To date, however, the published experience with NightWare is confined to military and veteran populations leaving its effectiveness among civilian first responders unknown.

Unlike military service, where deployment-related trauma may occur during discrete periods, first responders often remain exposed to repeated traumatic incidents throughout their careers while continuing to work, sleep, and return to duty in the same communities where the events occurred. First responders share much of the trauma and sleep-disturbance profile of military and veteran populations but differ in important ways, including ongoing exposure to repeated traumatic events throughout their careers, fitness-for-duty requirements, return-to-work expectations, organizational culture, and barriers to seeking behavioral health care. These differences may influence both treatment needs and treatment response, making it inappropriate to assume that findings from military populations generalize directly to civilian public safety personnel. We therefore evaluated the real-world response to NightWare over 60 days among first responders treated within a San Francisco program.

## Methods

### Design and participants

This was a single-arm, open-label, prospective evaluation of first responders (law enforcement and fire service personnel) in San Francisco who were prescribed NightWare as part of routine clinical care for trauma-related nightmares or nightmare disorder, consistent with the device's authorized indication. Participants completed the same self-report survey at device initiation (Day 0/baseline), Day 30, and 60. Eleven individuals initiated treatment and provided baseline data; one did not complete any follow-up assessment and was recorded as a single loss to follow-up, yielding a complete cohort of ten. Systematic demographic, comorbidity, and concomitant-treatment data were not captured as part of this evaluation.

### Selection criteria and demographics

The requirement for participation was to be a first responder over the age of 22 with at least 3 nightmares per week. The participants were selected from San Francisco Police and Fire Departments; half of the participants were policemen and half were firemen. There were 4 active duty service members and 6 service members that were either on leave mandated by workman's comp or fully retired. A PTSD diagnosis was not a requirement, but all participants did have PTSD. The population was split between 5 female and 5 male participants. There were 2 people aged 35–40. There were 5 people aged 41–50. There were 2 people aged 51–60, and 1 person over the age of 60. These patients were undergoing mental health therapy of various frequencies, but this therapy was not a requirement for participation. Medication information was not collected, but patients were requested to be on a stable dose of any medication they were taking for at least 2 weeks prior to participation. None of the participants were taking prazosin during the study. No other exclusion criteria were applied during this study.

### Intervention

NightWare comprises an Apple Watch and companion iOS application. During sleep, it continuously estimates a physiologic stress index from heart rate and movement, learns the individual's pattern, and when arousal consistent with an emerging nightmare is detected delivers brief, automatically calibrated vibrations intended to interrupt the nightmare while aiming not to awaken the user. The device was prescribed and used on-label, and patients were instructed to wear it nightly.

### Onboarding

Patients were contacted for onboarding and verbal consent to gather de-identified data and use it as needed, including for publication. During the onboarding call, patients were walked through how to turn on the watch to access the Nightware app. They were informed of the calibration period, educated on interventions, and best use tips. Participants were asked to wear the device at least 50% of the time, as the more they wear it, the better it will work for them. Participants were informed of the intended treatment effect of the NightWare device. These effects include perceived decrease in nightmare frequency and decreased distress from nightmares. A brief survey was given before the call ended to gather baseline data. Patients were told they will be contacted after 30 and 60 days to gather remaining feedback.

### Outcome measures

Outcomes were captured with a patient-reported survey developed in collaboration with the U.S. Department of Veterans Affairs (Veteran Affairs Veteran Health Administration Innovation Ecosystem, Washington D.C) for innovation pilots. The two co-primary endpoints were (1) nightmare frequency self-reported number of nights with nightmares in the prior week (integer, 0–7 days per week); and (2) nightmare-related distress, “How much distress have you had because of nightmares in the past week?”—on a 5-point ordinal scale (0-not at all distressed, 1-a little distressed, 2-moderately distressed, 3-quite a bit distressed, 4-extremely distressed). Secondary measures were overall sleep quality on an ordinal 5-point scale (1-very bad, 2-fairly bad, 3-neither good nor bad, 4-fairly good, 5-very good) and treatment satisfaction on an ordinal 5-point scale (1-very dissatisfied, 2-somewhat dissatisfied, 3-neither satisfied or dissatisfied, 4-somewhat satisfied, 5-very satisfied). Treatment satisfaction was only measured at Day 0 and Day 60. All other measures were measured at Day 0, Day 30, and Day 60. This instrument is fit-for-purpose for a pilot but is not an independently validated psychometric scale. Although the instrument has not been validated, it asks about what Nightware intends to accomplish- nightmare frequency and severity reduction.

### Statistical analysis

Each co-primary endpoint was treated as a continuous measure and analyzed with standard parametric methods for paired data. Change from baseline to Day 60 was assessed with a paired (dependent-samples) *t*-test, reporting the mean within-subject change, its 95% confidence interval, and Cohen's d as the standardized effect size (0.2 small, 0.5 medium, 0.8 large). The trend across the three time points (Day 0/30/60) was assessed with one-way repeated-measures analysis of variance (RM-ANOVA), with partial η^2^ as the effect size. The normality of the within-subject differences was checked with the Shapiro–Wilk test and was satisfied for both endpoints (*p* = 0.14 and *p* = 0.17). As a sensitivity analysis appropriate to the small sample and the ordinal outcome scales, the change from baseline to Day 60 in each endpoint was re-examined with the exact Wilcoxon signed-rank test, the trend across the three assessments with the Friedman test (Kendall's W as the effect size), and the median change with the Hodges–Lehmann estimator and its distribution-free 95% confidence interval.

Prespecified responder analyses summarized the proportions achieving any reduction and ≥50% reduction in nightmare frequency, and improvement of ≥1 and ≥2 distress categories. All tests were two-sided at α = 0.05; given the open-label, single-arm design and *n* = 10, *p*-values are interpreted as exploratory and hypothesis-generating. Missing data were handled by complete-case analysis. Analyses were performed in Python 3 (SciPy). Secondary endpoints did not undergo statistical analysis; simple means were calculated for these endpoints.

### Ethics

This work was conducted as a quality-improvement/program evaluation of routine clinical care and was exempt from institutional review board review. Data were de-identified for analysis. The evaluation was conducted in accordance with the principles of the Declaration of Helsinki.

## Results

### Patient adherence to treatment

The participants in this survey used the NightWare device a mean of 76% of nights (45.6 days of the 60-day survey time period). The range of usage was 55–100% (33–60 days) of the study nights. This amount of usage is expected to result in a decrease in nightmare frequency and severity, based on previous reported data in a military veteran population (Davenport and Werner, 2023).

### Patient response

**Figure 1:**
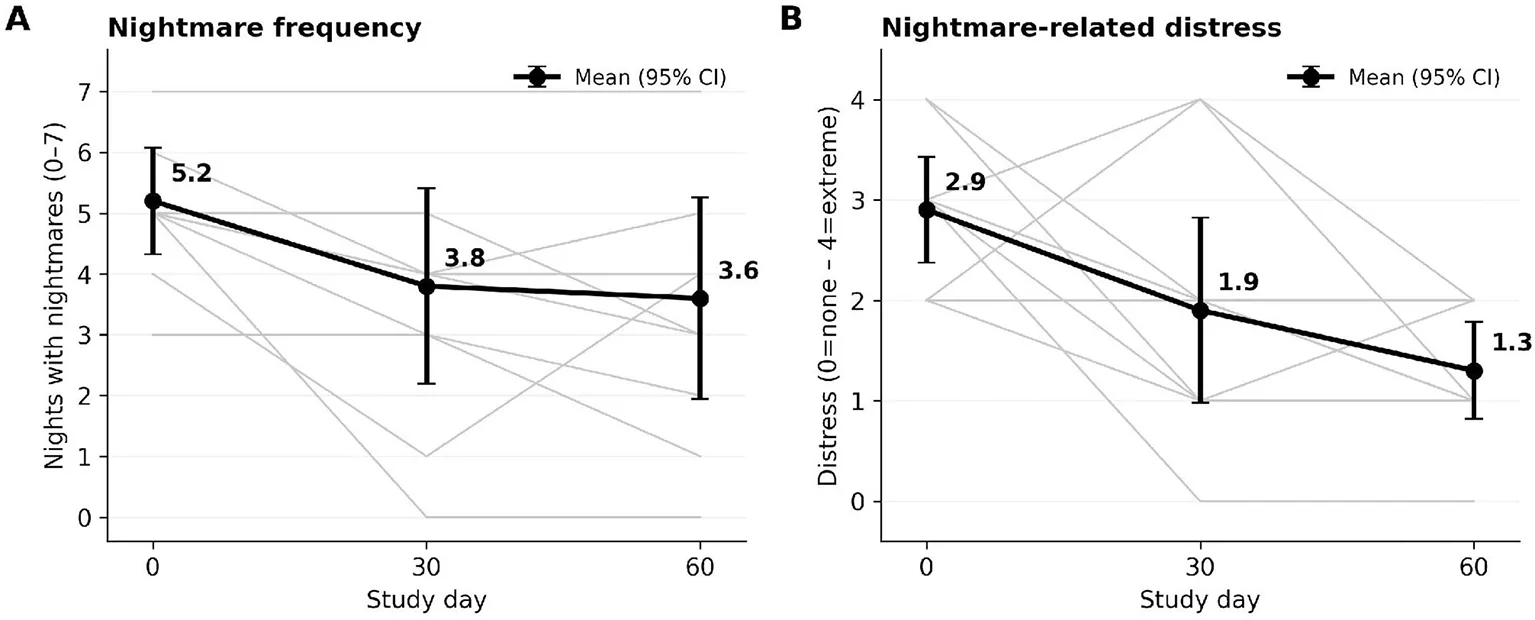
Individual participant trajectories (gray) and group mean with 95% confidence interval (black) for **(A)** nightmare frequency (nights/week) and **(B)** nightmare-related distress (0–4) at Day 0, 30, and 60 (*n* = 10 completers). No participant worsened on either measure from baseline to Day 60.

Of 11 first responders who initiated NightWare and provided baseline data, 10 (91%) completed all three assessments; one was lost to follow-up after baseline. The cohort was symptomatic at baseline, reporting a mean of 5.2 nights with nightmares per week and a mean distress rating of 2.9 (between “moderately” and “quite a bit”). Both co-primary endpoints improved over the 60-day evaluation, and no participant worsened on either measure from baseline to Day 60 (see Figure 1 and Table 1).

**Table 1:** Co-primary outcomes across time points and change from baseline to Day 60 (*n* = 10 completers).

Outcome (mean ±SD)	Day 0	Day 30	Day 60	Day 0 → 60 change (paired *t*-test)
Nightmare frequency (nights/wk)	5.2 ± 1.2	3.8 ± 2.3	3.6 ± 2.3	−1.6 (95% CI −2.7, −0.5); *p* = 0.011; *d* = 1.0
Nightmare distress (0–4)	2.9 ± 0.7	1.9 ± 1.3	1.3 ± 0.7	−1.6 (95% CI −2.2, −1.0); *p* = 0.0002; *d* = 1.9

RM-ANOVA across Day 0/30/60: frequency *F*_(2, 18)_ = 6.18, *p* = 0.009, partial η^2^ = 0.41; distress *F*_(2, 18)_ = 8.24, *p* = 0.003, partial η^2^ = 0.48. Within-subject differences were consistent with normality (Shapiro–Wilk *p* = 0.14 and 0.17).

Nightmare frequency decreased from a mean of 5.2 (SD 1.2) nights/week at baseline to 3.8 (SD 2.3) at Day 30 and 3.6 (SD 2.3) at Day 60. The mean within-subject change from baseline to Day 60 was 1.6 nights/week [95% CI −2.7 to −0.5; *t*_(9)_ = −3.21, *p* = 0.011], a large effect (Cohen's *d* = 1.0); RM-ANOVA confirmed a significant decline across the three time points [*F*_(2, 18)_ = 6.18, *p* = 0.009; partial η^2^ = 0.41]. Nightmare-related distress decreased from a mean of 2.9 (SD 0.7) at baseline to 1.9 (SD 1.3) at Day 30 and 1.3 (SD 0.7) at Day 60. The mean change from baseline to Day 60 was −1.6 points [95% CI −2.2 to −1.0; *t*_(9)_ = −6.00, *p* = 0.0002], a large effect (Cohen's *d* = 1.9); RM-ANOVA again confirmed a significant decline [*F*_(2, 18)_ = 8.24, *p* = 0.003; partial η^2^ = 0.48]. Most of the improvement in nightmare frequency was evident by Day 30 and was maintained through Day 60, whereas the clearest separation in distress emerged by Day 60.

### Responder analysis

Seven of ten patients (70%) reduced their nightmare frequency from baseline to Day 60, including three (30%) with a reduction of at least 50%; the remaining three were unchanged and none increased. On distress, nine of ten (90%) improved by at least one category and six (60%) improved by at least two categories; none worsened (see Table 2).

**Table 2:** Responder proportions, Day 0 to 60 (*n* = 10).

Definition	*n*/10	%
Any reduction in nightmare frequency	7	70%
≥50% reduction in nightmare frequency	3	30%
Distress improved ≥1 category	9	90%
Distress improved ≥2 categories	6	60%

### Sensitivity analysis

The non-parametric sensitivity analysis was concordant with the primary parametric analysis across all endpoints. From baseline to Day 60, nightmare frequency decreased significantly (Wilcoxon exact *p* = 0.016; Hodges–Lehmann median change −1.5 nights/week, 95% CI −2.5 to −0.5) and nightmare-related distress decreased significantly (*p* = 0.004; −1.5 points, 95% CI −2.0 to −1.0), while self-reported sleep quality increased significantly (*p* = 0.004; + 2.0 points, 95% CI + 1.5 to + 3.0). The Friedman test confirmed significant change across the three assessments for all endpoints (frequency *p* = 0.007, *W* = 0.49; distress *p* = 0.005, *W* = 0.54; sleep quality *p* < 0.001, *W* = 0.74). Full results appear in Supplementary Table S1.

### Secondary measures

Self-reported overall sleep quality improved over the evaluation period (mean rating 1.8 at Day 0, 3.2 at Day 30, and 4.0 at Day 60). Treatment satisfaction also improved [mean rating 2.2 at Day 0 (satisfaction with current/prior treatment), and 4.5 at Day 60 (satisfaction with Nightware as treatment)]. Nine of the 10 participants elected to continue use of the NightWare device after the pilot period.

## Discussion

This pilot captures real-world responses to NightWare among first responders—a population with a high burden of occupational trauma, PTSD, and nightmares but no prior published experience with the device. Over 60 days, treatment was associated with large, statistically apparent, and internally consistent reductions in both nightmare frequency and nightmare-related distress, with improvement on two distinct endpoints, no worsening in any participant, and high patient satisfaction. The direction and magnitude of these findings are consistent with the existing military and veteran literature and extend that experience to the first-responder setting.

This study, and the study in veterans (Davenport and Werner, 2023), both demonstrate that treatment adherence is crucial to the efficacy of the device. As with other therapies, patient selection is likely essential to successful treatment, as the decrease in nightmare distress and frequency will reinforce use of the device. Because NightWare relies on changes in heart rate and movement to detect when nightmares are most likely occurring, patients with pronounced sympathetic-arousal features (for example, awakening with racing heart, sweating, or panic-like symptoms) may be the most appropriate candidates (Robertson et al., 2023). Our cohort was clearly symptomatic at baseline, which may have contributed to the consistency of the observed response. These results align with the randomized, sham-controlled trial, in which the active device outperformed sham among participants who used it regularly (Davenport and Werner, 2023), and with the large real-world series in active-duty service members, in which the great majority reported improvement with good tolerability (Robertson et al., 2023).

NightWare may help address well-recognized gaps in nightmare care. Imagery rehearsal therapy is effective in some patients but limited by a shortage of trained providers and by patients who are unable or unwilling to engage in it (Morgenthaler et al., 2018; Cook et al., 2010), and pharmacologic options such as prazosin are limited by inconsistent efficacy and side effects (El-Solh, 2018). For first responders in particular, fitness-for-duty and occupational requirements can further restrict the use of centrally acting medications. A non-pharmacologic, wearable intervention that is simple to use, low in risk, and scalable with little demand on clinician time is therefore an appealing addition to the treatment armamentarium for this workforce and may also offer privacy to those who perceive stigma in seeking behavioral health care.

The public-health stakes of untreated nightmares in first responders are considerable, given the documented associations among nightmares, disturbed sleep, and suicide and the elevated suicide burden in this population (Stanley et al., 2016; Tanskanen et al., 2001). We reiterate, however, that this evaluation neither measured nor can speak to suicidality or any outcome beyond the self-reported sleep measures collected; NightWare is not indicated for the reduction of suicide risk, and treating nightmare disorder should be regarded at most as one component of comprehensive, multidisciplinary care. Nightmares are among the most disabling residual symptoms of trauma experienced by first responders. Beyond disrupting sleep, they often contribute to anticipatory anxiety about going to bed, emotional exhaustion, relationship strain, and reduced occupational readiness. Interventions that reduce nightmare burden therefore have the potential to improve both psychological functioning and operational performance.

This study has important limitations. It was a small, single-arm, open-label evaluation without a control group, so improvement cannot be attributed to the device alone; regression to the mean, natural symptom fluctuation, expectation effects, and the attention of a monitored program may all have contributed. The sample was small (*n* = 10), making estimates imprecise and sensitive to individual participants. Outcomes were self-reported using a bespoke, non-validated instrument, and the distress endpoint is an ordinal item analyzed as a continuous score; the secondary sleep-quality and satisfaction measures changed in form or referent across visits and are reported descriptively. No objective sleep data, demographics, comorbidities, or concomitant treatments were captured. Finally, the evaluation was company-affiliated (see Conflict of interest). These constraints place the findings firmly in the hypothesis-generating category.

## Conclusion

In this 60-day open-label pilot, treatment with NightWare was associated with reductions in nightmare frequency and distress and high acceptability among San Francisco first responders—an under-studied population that carries a substantial trauma and sleep-disturbance burden. Although additional well-controlled studies with validated instruments are needed, these real-world results are consistent with the existing military and veteran experience and support further investigation of NightWare in first responders.

## Data Availability

The original contributions presented in the study are included in the article/Supplementary material, further inquiries can be directed to the corresponding author.
