# Supplementary material for: Real-world reduction in nightmare frequency and distress among San Francisco first responders treated with a prescription wearable device: a 60-day open-label pilot
Source: Front Sleep. 2026 Sep 9;5:1941077. doi: 10.3389/frsle.2026.1941077 (PMC13597376; doi:10.3389/frsle.2026.1941077)
Supplement: Supplementary file 1 [file Table_1.docx]

# **Results — primary (parametric) vs. sensitivity (non-parametric)**

**Supplementary Table S1.** Change from baseline to Day 60 for each within-subject endpoint under the primary parametric analysis and the non-parametric sensitivity analysis (n=10 completers).

| **Endpoint** | **Primary analysis (parametric)** | **Sensitivity analysis (non-parametric)** |
| --- | --- | --- |
| **Nightmare frequency** (nights/wk; ↓ better) | Mean Δ −1.6 (95% CI −2.7, −0.5); paired t(9)=−3.21, p=0.011; d=1.0. RM-ANOVA F(2,18)=6.18, p=0.009, η²ₚ=0.41. | Wilcoxon signed-rank exact p=0.016; Hodges–Lehmann median Δ −1.5 (95% CI −2.5, −0.5). Friedman p=0.007, Kendall’s W=0.49. |
| **Nightmare distress** (0–4 ordinal; ↓ better) | Mean Δ −1.6 (95% CI −2.2, −1.0); paired t(9)=−6.00, p=0.0002; d=1.9. RM-ANOVA F(2,18)=8.24, p=0.003, η²ₚ=0.48. | Wilcoxon signed-rank exact p=0.004; Hodges–Lehmann median Δ −1.5 (95% CI −2.0, −1.0). Friedman p=0.005, Kendall’s W=0.54. |
| **Sleep quality** (1–5 ordinal; ↑ better) | Mean Δ +2.2 (95% CI +1.4, +3.0); paired t(9)=6.13, p=0.0002; d=1.9. RM-ANOVA F(2,18)=26.16, p<0.001, η²ₚ=0.74. | Wilcoxon signed-rank exact p=0.004; Hodges–Lehmann median Δ +2.0 (95% CI +1.5, +3.0). Friedman p<0.001, Kendall’s W=0.74. |

*Effect-size guide: Cohen’s d and Kendall’s W — 0.1/0.2 small, 0.3/0.5 medium, 0.5/0.8 large. All endpoints show large effects under both approaches. Responders (baseline→Day 60): frequency 7/10 improved, 0 worsened; distress 9/10 improved, 0 worsened; sleep quality 9/10 improved, 0 worsened.*
